# Depletion of alveolar macrophages in CD11c diphtheria toxin receptor mice produces an inflammatory response

**DOI:** 10.1002/iid3.51

**Published:** 2015-03-23

**Authors:** Lydia M Roberts, Hannah E Ledvina, Shraddha Tuladhar, Deepa Rana, Shaun P Steele, Gregory D Sempowski, Jeffrey A Frelinger

**Affiliations:** 1 Department of Immunobiology, University of ArizonaTucson, Arizona, USA; 2 Department of Microbiology and Immunology, University of North Carolina at Chapel HillChapel Hill, North Carolina, USA; 3 Duke Human Vaccine InstituteDurham, North Carolina, USA

**Keywords:** Alveolar macrophage, animal models, CD11c diphtheria toxin receptor mice, lung, neutrophil

## Abstract

Alveolar macrophages play a critical role in initiating the immune response to inhaled pathogens and have been shown to be the first cell type infected following intranasal inoculation with several pathogens, including *Francisella tularensis*. In an attempt to further dissect the role of alveolar macrophages in the immune response to *Francisella*, we selectively depleted alveolar macrophages using CD11c.DOG mice. CD11c.DOG mice express the diphtheria toxin receptor (DTR) under control of the full CD11c promoter. Because mice do not express DTR, tissue restricted expression of the primate DTR followed by treatment with diphtheria toxin (DT) has been widely used as a tool in immunology to examine the effect of acute depletion of a specific immune subset following normal development. We successfully depleted alveolar macrophages via intranasal administration of DT. However, alveolar macrophage depletion was accompanied by many other changes to the cellular composition and cytokine/chemokine milieu in the lung that potentially impact innate and adaptive immune responses. Importantly, we observed a transient influx of neutrophils in the lung and spleen. Our experience serves as a cautionary note to other researchers using DTR mice given the complex changes that occur following DT treatment that must be taken into account when analyzing data.

## Introduction

Alveolar macrophages are spatially poised to be the first immune cells that interact with inhaled pathogens. In addition to our work with *F. tularensis* demonstrating alveolar macrophages are initially infected after intranasal inoculation, other groups have demonstrated that *Mycobacterium tuberculosis*, *Mycoplasma pulmonis*, and *Legionella pneumophila* infect alveolar macrophages 1–6. Not only do alveolar macrophages serve as an important innate immune cell to combat infection, they are also a bridge between the innate and adaptive immune response via production of cytokines that shape the T cell response. In several infection models, removal of alveolar macrophages significantly increases pathogen burdens and the host response is associated with exacerbated inflammation 7–10.

To elucidate the role of alveolar macrophages during *F. tularensis* infection, we used an approach adopted widely to specifically ablate particular cell populations via diphtheria toxin-mediated killing 11–17. Heparin-binding EGF-like growth factor precursor is the diphtheria toxin receptor (DTR) in primates; but, the mouse form of this molecule does not bind diphtheria toxin 18. This allows the generation of transgenic mice where expression of the DTR is controlled by a cell-type specific promoter 19. However, several groups have described limitations of DTR mice, including promiscuous expression of the DTR which causes mouse death when repeated injections of diphtheria toxin (DT) are given 13,20. A newer DTR mouse called CD11c.DOG utilizes a bacterial artificial chromosome with the cloned DTR under control of the full CD11c promoter 17. This mouse tolerates repeated injections of DT unlike previous iterations 17.

Using the CD11c.DOG system, we sought to remove alveolar macrophages from the lung via administration of DT in order to study the role of this cell type during *Francisella* infection. Because the lung is accessible without systemic treatment, we hypothesized that administration of a low dose of DT intranasally would selectively deplete alveolar macrophages in the lung while leaving systemic dendritic cells intact. Tittel et al. reported that systemic DT treatment of CD11c.DOG mice results in polymorphonuclear neutrophil (PMN) release from the bone marrow, which in turn causes chemokine-dependent neutrophilia 21. We hypothesized that a low dose of DT delivered directly to the lung would not have the same effect as systemic DT treatment in terms of subsequent PMN influx. However, intranasal DT treatment did induce an influx of neutrophils to the lung that dissipated by day 5 post-DT treatment. Although alveolar macrophages remained depleted at this time point, we observed changes in the cell composition of the lung as well as cytokine and chemokine milieu following DT treatment that made it very difficult to draw conclusions on the role of alveolar macrophages during an inflammatory process. Therefore, we emphasize that DTR-mediated depletion, even when DT is given locally, does not simply affect one cell type and instead introduces many confounding effects. Furthermore, any conclusions made from experiments using DTR transgenic mice need to carefully consider the immunological changes that occur upon DT treatment.

## Results

### Intranasal Diphtheria Toxin Treatment Depletes Alveolar Macrophages

**Figure 1:**
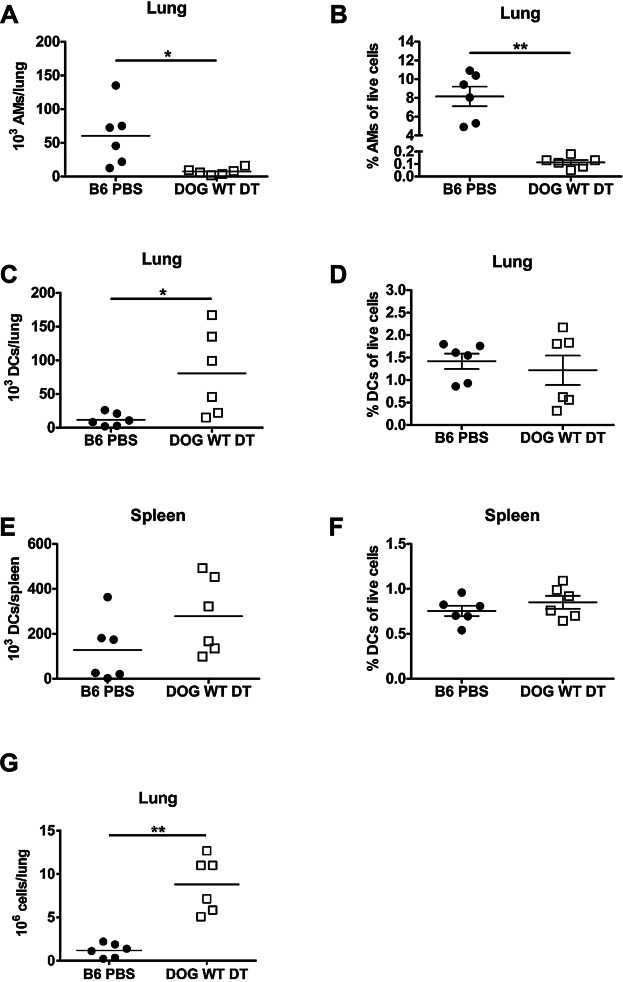
Intranasal diphtheria toxin treatment depletes alveolar macrophages. C57Bl/6J mice were intranasally inoculated with 50 µL PBS (B6 PBS) or CD11c.DOG mice were intranasally inoculated with 8 ng of diphtheria toxin in 50 µL PBS (DOG WT DT). Twenty-four hours later, lungs and spleens were removed, made into single cell suspensions, and stained for flow cytometry analysis. The A) absolute number of alveolar macrophages (AMs), B) % AMs of live cells, C) absolute number of lung dendritic cells (DCs), D) % lung DCs of live cells, E) absolute number of spleen DCs, and F) % spleen DCs of live cells was determined using flow cytometry. G) The total number of live lung cells was determined using a hemacytometer. n = 6 mice/group. Statistical significance was determined using a Mann-Whitney test. Data are representative of 3 independent experiments.

Alveolar macrophages are spatially positioned in the alveolar space of the lung and are therefore the first immune cells to interact with inhaled pathogens. We found that *Francisella tularensis* live vaccine strain (LVS) initially infects alveolar macrophages following intranasal inoculation 1. We therefore sought to determine whether bacterial clearance was altered in the absence of alveolar macrophages. Alveolar macrophages express high levels of CD11c 1,22 thereby allowing us to utilize the CD11c.DOG mouse to selectively deplete alveolar macrophages via intranasal inoculation with diphtheria toxin. We hypothesized that local treatment with diphtheria toxin (DT) would remove alveolar macrophages selectively and preserve peripheral dendritic cells since DT is poorly absorbed through the lung. Thus, the effective DT dose in the periphery is very low. We selected a lower intranasal dose of DT (8 ng) compared to other published studies, which used 50–100 ng, because we wished to selectively deplete alveolar macrophages while leaving lung dendritic cells intact 23–25. Furthermore, we hypothesized that direct inoculation with a low dose of DT would not result in a neutrophilic influx that has previously been observed with systemic DT treatment 21. Indeed, intranasal DT treatment of CD11c.DOG mice depleted alveolar macrophages in the lungs compared to PBS treated B6 mice (Fig. 1A, B), but left both lung and spleen dendritic cell populations intact (Fig. 1C–F). We observed a significant increase in the absolute number of lung dendritic cells (Fig. 1C), but there was no difference in the frequency of dendritic cells (Fig. 1D) indicating the increase in total lung cellularity (Fig. 1G) is driving the significant increase in the absolute number of lung dendritic cells.

### Diphtheria Toxin Treatment Increases Systemic Neutrophils

**Figure 2:**
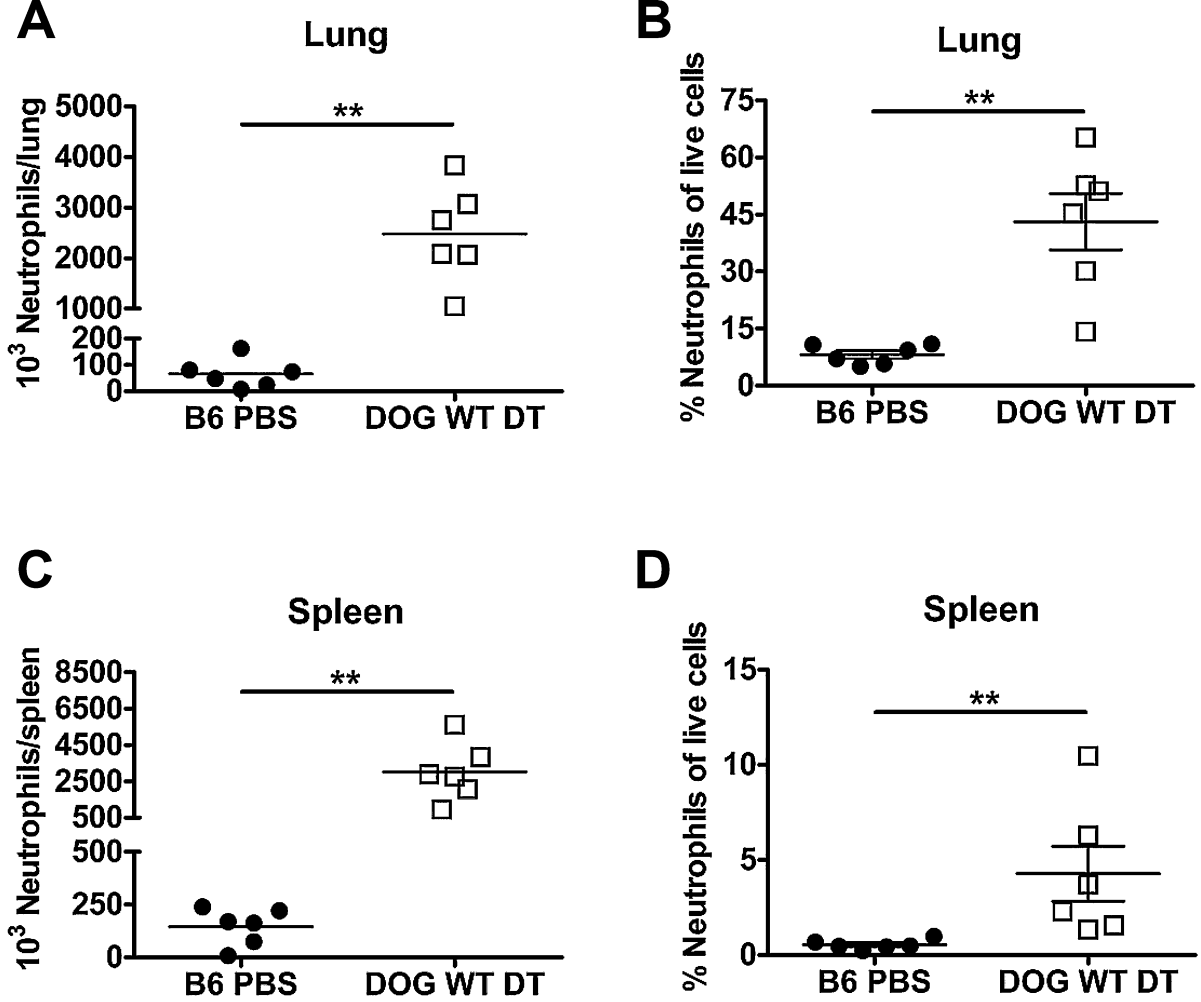
Intranasal diphtheria toxin treatment increases systemic neutrophils 24 hours after administration. C57Bl/6J mice were intranasally inoculated with 50 µL PBS (B6 PBS) or CD11c.DOG mice were intranasally inoculated with 8 ng of diphtheria toxin in 50 µL PBS (DOG WT DT). 24 hours later, lungs and spleens were removed, made into single cell suspensions, and stained for flow cytometry analysis. The A) absolute number of lung neutrophils, B) % lung neutrophils of live cells, C) absolute number of spleen neutrophils, and D) % spleen neutrophils of live cells was determined using flow cytometry. n = 6 mice/group. Statistical significance was determined using a Mann-Whitney test. Data are representative of 3 independent experiments.

As unmanipulated B6 mice inoculated intranasally with 500 CFU of the LVS survive and clear their infection, we were surprised to find that CD11c.DOG mice treated with DT and intranasally inoculated 24 hours later with LVS died within 72 hours from a neutrophilic pneumonia (data not shown). We used flow cytometry to examine the cells recovered from the lungs of uninfected, but DT treated CD11c.DOG mice 24 hours after DT inoculation and found a large neutrophil infiltrate in both the lung and spleen that was significantly greater than in PBS treated B6 mice (Fig. 2). These data indicate that neutrophilic influx occurs with a local, low-dose inoculation with DT and not only when DT is given systemically. We therefore decided to let the mice rest for 5 days after DT inoculation to allow time for the neutrophil infiltration to return to baseline given that neutrophils have a rapid turnover rate.

### Alveolar Macrophages Remain Depleted 5 Days After Intranasal Diphtheria Toxin Treatment

It was possible that the DT preparation was contaminated by molecules that might themselves be TLR/NLR ligands and these contaminants were mediating the response. Therefore, in addition to resting mice for 5 days, we added an additional control to determine whether changes in the cell composition of the lung was due to depletion of alveolar macrophages, or a consequence of innate receptor stimulation by contaminants in the DT. A glutamine substitution at position 52 renders DT enzymatically inactive 26, but the purification process from *Corynebacterium diphtheriae* is identical to wild-type DT (WT DT) allowing us to distinguish between events that occur as a consequence of cellular depletion or TLR stimulation.

**Figure 3:**
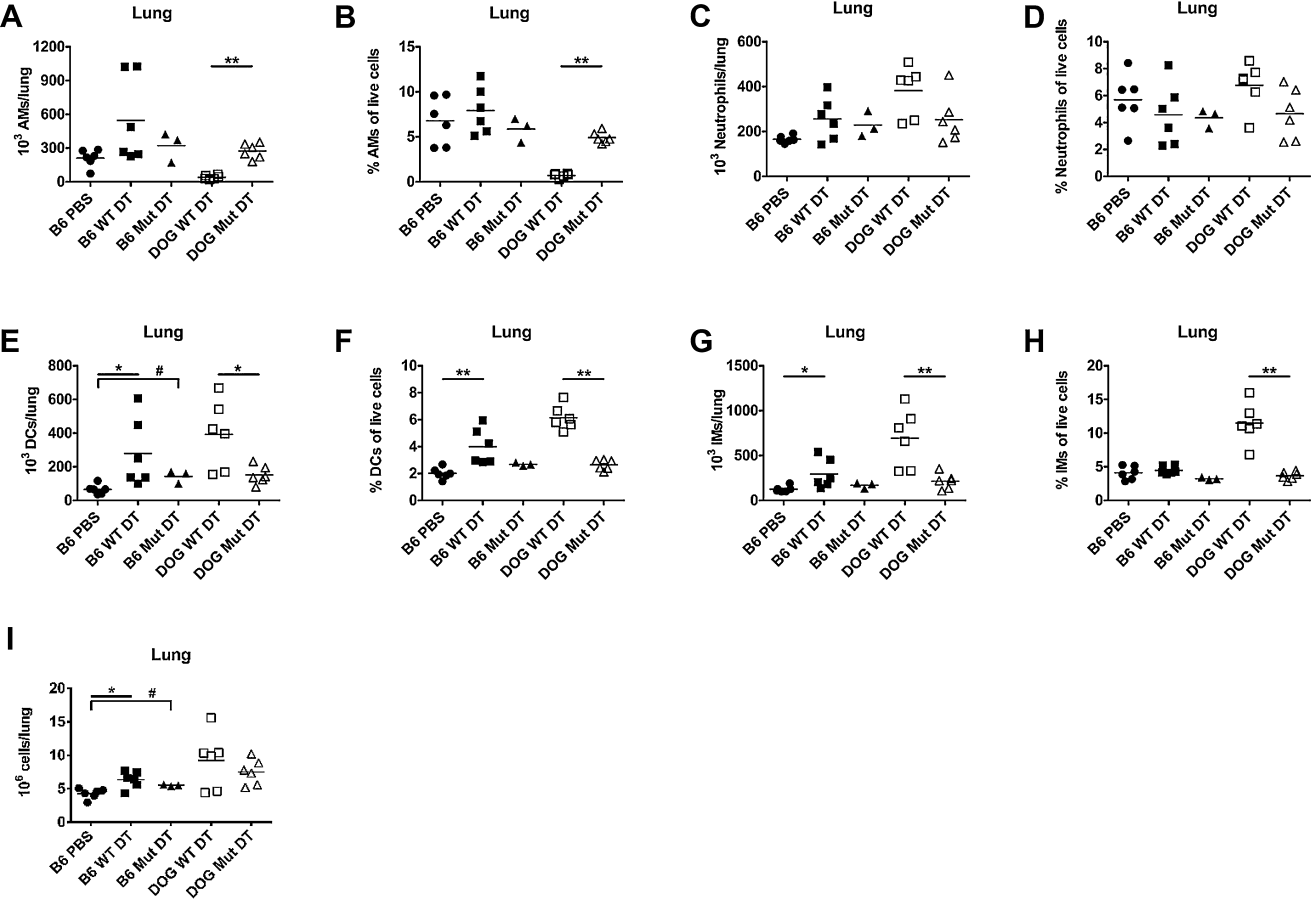
Alveolar macrophages are depleted from the lung 5 days after intranasal diphtheria toxin treatment. C57Bl/6J mice were intranasally inoculated with 50 µL PBS (B6 PBS), 8 ng wild-type diphtheria toxin (B6 WT DT) or 8 ng mutant diphtheria toxin (B6 Mut DT) or CD11c.DOG mice were intranasally inoculated with 8 ng of wild-type diphtheria toxin (DOG WT DT) or 8 ng mutant diphtheria toxin (DOG Mut DT) in 50 µL PBS. Five days later, lungs were removed, made into single cell suspensions, and stained for flow cytometry analysis. The A) absolute number of alveolar macrophages (AMs), B) % AMs of live cells, C) absolute number of lung neutrophils, D) % lung neutrophils of live cells, E) absolute number of lung dendritic cells (DCs), F) % lung DCs of live cells, G) absolute number of lung interstitial macrophages (IMs), and H) % lung IMs of live cells was determined using flow cytometry. I) The total number of lung cells was determined using a hemacytometer. n = 3-6 mice/group. An ANOVA with Tukey's post-test was used to determine whether the B6 groups were significantly different from each other different from each other. A Mann-Whitney test was used to determine if the pooled B6 groups were significantly different from the DOG Mut DT or if the DOG WT DT and DOG Mut DT were significantly different. Data are representative of at least 2 independent experiments.

Alveolar macrophages were still >90% depleted 5 days after WT DT treatment in CD11c.DOG mice (Fig. 3A, B). Importantly, alveolar macrophage depletion only occurred in CD11c.DOG mice treated with WT DT (Fig. 3A, B). Additionally, the absolute number and percentage of lung neutrophils in the WT DT-treated CD11c.DOG mice was the same as mutant DT-treated CD11c.DOG mice (Fig. 3C, D). Although neutrophils were unchanged in WT DT-treated CD11c.DOG mice, the absolute number and percentage of lung dendritic cells and interstitial macrophages were increased compared to mutant DT-treated CD11c.DOG mice (Fig. 3E–H). Giving the mice additional time to rest after DT treatment also eliminated the increase in lung cellularity observed at 24 hours (Fig. 1G) with no statistically significant increase in WT DT-treated CD11c.DOG compared to either WT DT-treated B6 mice or mutant DT-treated CD11c.DOG mice (Fig. 3I). Together, these data indicate that WT DT treatment alters the cell composition of the lung in addition to the removal of alveolar macrophages. Furthermore, since mutant DT inoculation of CD11c.DOG mice did not result in changes in the cell composition of the lung, we can conclude that the changes we see are a result of alveolar macrophage depletion and not contamination of the DT with TLR ligands.

**Figure 4:**
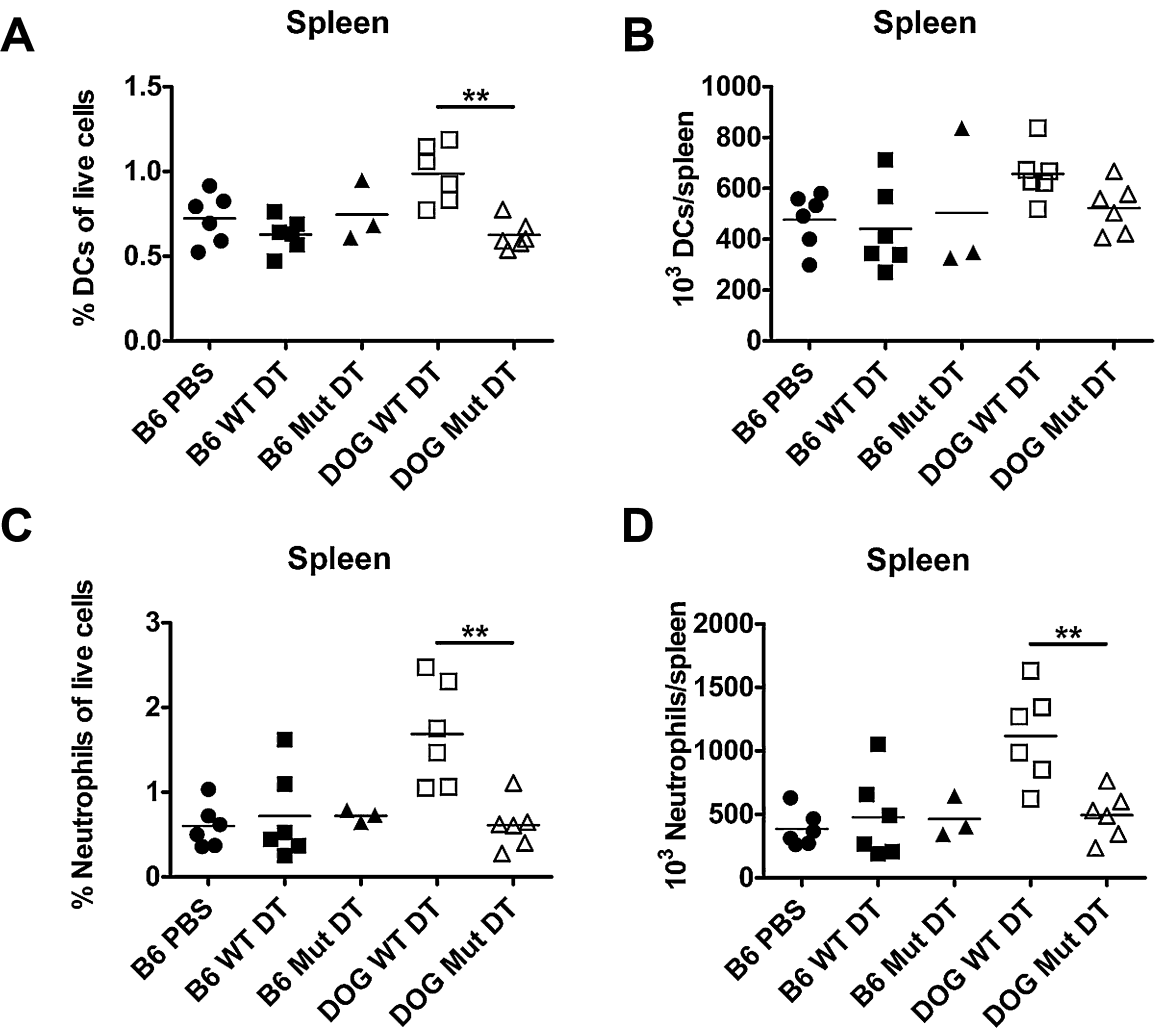
Diphtheria toxin treatment alters the spleen's cellular composition 5 days post-inoculation. C57Bl/6J mice were intranasally inoculated with 50 mL PBS (B6 PBS), 8 ng wild-type diphtheria toxin (B6 WT DT) or 8 ng mutant diphtheria toxin (B6 Mut DT) or CD11c.DOG mice were intranasally inoculated with 8 ng of wild-type diphtheria toxin (DOG WT DT) or 8 ng mutant diphtheria toxin (DOG Mut DT) in 50 µL PBS. Five days later, spleens were removed, made into single cell suspensions, and stained for flow cytometry analysis. The A) % spleen dendritic cells (DCs) of live cells, B) absolute number of spleen DCs, C) % spleen neutrophils of live cells, and D) absolute number of spleen neutrophils was determined using flow cytometry. n = 3-6 mice/group. An ANOVA with Tukey's post-test was used to determine whether the B6 groups were significantly different from each other. A Mann-Whitney test was used to determine if the pooled B6 groups were significantly different from the DOG Mut DT or if the DOG WT DT and DOG Mut DT were significantly different. Data are representative of at least 2 independent experiments.

The percentage of spleen dendritic cells was significantly increased in WT DT-treated CD11c.DOG mice compared to mutant DT-treated CD11c.DOG mice; however, there was no significant increase in the absolute number of dendritic cells (Fig. 4A, B). While the percentage of splenic neutrophils was still significantly increased 5 days after CD11c.DOG mice were inoculated with WT DT, the increase was only approximately 2-fold (Fig. 4C). In comparison, the percentage of neutrophils was approximately 8-fold higher at 24 hours post DT treatment (Fig. 2D). The increase in percentage of neutrophils was also accompanied by a significant increase in their absolute number (Fig. 4D). As in the lung, mutant DT had no effect on spleen neutrophil levels suggesting that neutrophilic influx was a consequence of alveolar macrophage depletion and did not result from TLR ligand contamination of the DT.

### The Lung Has a Pro-inflammatory Milieu Following Diphtheria Toxin Treatment

**Figure 5:**
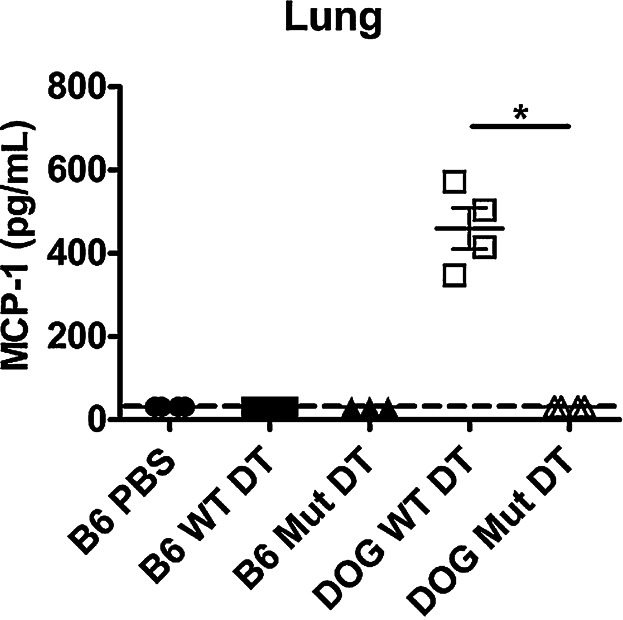
Diphtheria toxin treatment increases the lung's MCP-1 concentration. C57Bl/6J mice were intranasally inoculated with 50 mL PBS (B6 PBS), 8 ng wild-type diphtheria toxin (B6 WT DT) or 8 ng mutant diphtheria toxin (B6 Mut DT) or CD11c.DOG mice were intranasally inoculated with 8 ng of wild-type diphtheria toxin (DOG WT DT) or 8 ng mutant diphtheria toxin (DOG Mut DT) in 50 µL PBS. Five days later, lungs were removed and homogenized. Lung homogenates were clarified by centrifugation and the concentration of MCP-1 was determined by ELISA. The dashed line indicates the limit of detection (31.25 pg/mL). n = 3-4 mice/group. An ANOVA with Tukey's post-test was used to determine whether the B6 groups were significantly different from each other. A Mann-Whitney test was used to determine if the pooled B6 groups were significantly different from the DOG Mut DT or if the DOG WT DT and DOG Mut DT were significantly different. Data are representative of 2 independent experiments.

During our experiments with *Francisella* infection, we noticed there was a large increase in the MCP-1 (CCL-2) and GM-CSF concentration in lung culture supernatants (Table1), but not other cytokines and chemokines conventionally associated with inflammation (i.e., no change in IFN-γ, IL-1, TNF-α, etc.). Importantly, GM-CSF, IL-12, MCP-1, and VEGF were significantly increased in CD11c.DOG mice that had been treated with DT, but not infected with LVS compared to mock infected B6 mice suggesting the presence of these molecules was due to the DT treatment. Furthermore, the absence of inflammatory cytokines suggested to us that the changes we observed were not due to the lack of alveolar macrophages, but instead due to the destruction of alveolar macrophages by DT and were responsible for the subsequent neutrophil infiltrate. Because of the large change in MCP-1 observed by multiplex bead array, we examined levels of MCP-1 in lung homogenates by ELISA in CD11c.DOG mice that were treated with DT but not inoculated with *Francisella* (Fig. 5). MCP-1 levels were significantly increased in the lungs suggesting that this chemokine was responsible for the increase in dendritic cells observed in the lung on day 5 post-DT treatment and could be playing a role in the altered cytokine response that was independent of infection. Importantly, this increase was only observed in CD11c.DOG mice administered WT DT, again indicating that the DT itself is not activating innate immune sensors and that the inflammatory environment observed is due to alveolar macrophage depletion. Taken together, our data indicates that DT treatment of CD11c.DOG mice leads to a large, systemic neutrophilic influx, as well as local cytokine and chemokine increases. These changes are important confounding factors that should be addressed when analyzing data from DT depletion experiments.

**Table 1:** Fold change in cytokine and chemokine concentrations from lung culture supernatant *n* = 3−4 mice/group

	CD11c.DOG Mock-B6 Mock	CD11c.DOG LVS-CD11c.DOG Mock	B6 LVS-B6 Mock	CD11c.DOG LVS-B6 LVS
FGF basic	1.00	1.00	1.00	1.00
GM-CSF	4.82^^*^^*^^*^^	0.99	3.35^^*^^*^^	1.43
IFN-γ	1.00	1.00	1.00	1.00
IL-1α	1.00	1.00	1.00	1.00
IL-1β	1.00	1.00	1.00	1.00
IL-2	1.00	1.00	1.00	1.00
IL-4	1.00	1.00	1.00	1.00
IL-5	2.45	0.74	1.62	1.12
IL-6	1.38	1.03	1.11	1.28
IL-10	1.00	1.00	1.00	1.00
IL-12	5.35^*^	1.77	1.71	5.53 ^^*^^*^^*^^
IL-13	1.00	1.00	1.00	1.00
IL-17	1.00	1.00	1.00	1.00
IP-10	0.55	1.57	0.52	1.67
KC	1.12	0.94	0.99	1.06
MCP-1	2.10^*^	1.27	2.35^^*^^*^^	1.13
MIG	8.50	1.49	1.00	12.70^^*^^*^^
MIP-1α	1.00	1.00	1.00	1.00
TNF-α	1.00	1.00	1.00	1.00
VEGF	4.63^^*^^*^^	1.05	1.39	3.48^^*^^*^^

All CD11c.DOG mice were treated with 8 ng DT 5 days prior to inoculation with PBS (mock) or LVS; B6 mice were not treated with DT. Lungs were harvested 4 hours after PBS or LVS inoculation. The value in each column indicates the fold change in cytokine or chemokine concentration when the two groups listed as the column heading are compared. An ANOVA with Tukey's post-test was used to determine whether groups were significantly different from each other.

## Discussion

Because alveolar macrophages were initially infected following intranasal inoculation with LVS 1, we first sought to determine whether the course of disease was altered in the absence of alveolar macrophages. Alveolar macrophages can be depleted by intranasal administration of liposomal clodronate; however, this treatment is non-specific and depletes >90% of lung and airway antigen presenting cells including alveolar macrophages 27. Therefore, we instead chose to use CD11c.DOG mice treated with DT so that alveolar macrophages are specifically depleted while other phagocytic cells remain untouched.

Diphtheria toxin (DT) has been used in many studies to selectively deplete cells from transgenic mice expressing the DTR under the control of tissue restricted promoters 11–17. We hypothesized that transgenic mice expressing DTR under the control of the CD11c promoter would be susceptible to ablation via intranasal DT without systemic effects on the dendritic cell population because alveolar macrophages are present near the surface of the lung epithelium. We found that alveolar macrophages were effectively depleted from the airway upon DT treatment, while both pulmonary and systemic dendritic cells were preserved. We showed that nearly all alveolar macrophages were depleted 24 hours after intranasal DT inoculation. Surprisingly, when mice were inoculated with LVS 24 hours post-DT treatment, they died rapidly of a neutrophilic pneumonia, well before even MyD88 knock-out mice infected with similar doses of *Francisella* succumb to infection 28,29. When we examined our flow cytometry data, we found neutrophils were significantly increased after DT treatment without *Francisella* infection. Neutrophils comprised nearly 50% of all live cells recovered from the lung. Thus, neutrophils may be recruited to the lung to remove the cellular debris that accumulates after alveolar macrophages have been removed by DT. Neutrophils were also increased in the spleen 24 hours after intranasal DT treatment indicating a systemic effect of the DT treatment. Tittel et al. reported systemic neutrophilia in CD11c.DTR and CD11c.DOG mice after intraperitoneal injection of DT (up to 40 ng/kg) 21. Other groups have also reported a transient increase in neutrophils after administration of 8-10 ng/g DT 17,30. To avoid the increase in lung neutrophils, we hypothesized that waiting 5 days after DT treatment would allow the neutrophils to dissipate (due to rapid cellular turnover) while alveolar macrophages would remain depleted because they exhibit slower cellular turnover. At this time point, alveolar macrophages were still >90% depleted while neutrophil levels had returned to baseline. However, there was a significant increase in the percentage of interstitial macrophages and dendritic cells in the lung on day 5 post-DT treatment, indicating that depletion of alveolar macrophages affected many populations within the lung.

Interestingly, some of the cytokines that were elevated likely aid in differentiating alveolar macrophages. For example, GM-CSF is necessary for expressing high levels of CD11c and is therefore likely involved in the re-population of lung alveolar macrophages 31. GM-CSF is also required for development of alveolar macrophages from fetal monocytes 32. Additional flow cytometry analysis of the lung on days 7 and 9 post-DT treatment indicated the alveolar macrophage population was returning (data not shown). These data suggest that increased GM-CSF and possibly other chemokines that were elevated contribute to re-populating the lung with alveolar macrophages. The cytokine profile following alveolar macrophage depletion suggests that the lung is primed to replenish this cell type. Furthermore, these data indicate that there is a feedback mechanism present in the lung that is capable of recognizing the depletion of a specific cell population and then driving the re-population of the missing cell type via the production of necessary chemokines and growth factors. These data may be relevant for the depletion of other cell types in other organs and should be monitored and controlled for appropriately when using DTR transgenic mice to study the role of specific cell populations.

Overall, these data demonstrate that there are confounding effects of what initially appeared to be a specific method to acutely ablate a single population of cells in a specific organ by combining selective exposure with restricted expression of DT. This system proved more complex than originally anticipated, as the death of a critical cell population fundamentally changed the environment of the immune response. The multitude of changes after DT treatment rendered it impossible to effectively differentiate differences seen in the immune response due to the loss of alveolar macrophages and the change in cytokine/chemokine milieu. Therefore, future studies involving DTR mice should take into account the complex changes that occur to the immune system when drawing conclusions from experimental data.

## Materials and Methods

### Mice

C57Bl/6J (B6) mice were obtained from The Jackson Laboratory (Bar Harbor, ME). B6-*Tg(Itgax-DTR/OVA/EGFP)*^1Garbi^ (CD11c.DOG) mice 17 were obtained from Gunter Hammerling (German Cancer Research Center, Heidelberg, Germany) and then bred in-house by backcrossing to B6 mice. CD11c.DOG mice were used at heterozygotes. All mice were housed in specific-pathogen free conditions at the University of Arizona in accordance with the Institutional Animal Care and Use Committee. Male and female mice used for experiments were between 7 and 12 weeks of age and were age and sex-matched with controls.

### Inoculation of Mice With Diphtheria Toxin

CD11c.DOG mice were anesthetized with 0.25 mL of 7.5 mg/mL ketamine and 0.5 mg/mL xylazine cocktail in PBS administered intraperitoneally. Mice were then intranasally inoculated with 8 ng of wild-type (WT) or enzymatically-inactive mutant (G52E) diphtheria toxin 26 (Sigma, St. Louis, MO) in 50 μL PBS.

### Single Cell Suspension of Mouse Spleen and Lung

Spleens were aseptically removed and made into single cell suspensions. Lungs were perfused with PBS, aseptically removed, and digested into a single cell suspension as previously described 33. Red blood cells were lysed using ammonium chloride potassium lysis buffer (Gibco) and washed with RPMI 1640 supplemented with 10% fetal calf serum (Atlas), L-glutamine, sodium pyruvate, and β-mercaptoethanol. The total number of viable cells was determined using a hemocytometer by trypan blue exclusion.

### Identification of Spleen and Lung Populations by Flow Cytometry

**Figure 6:**
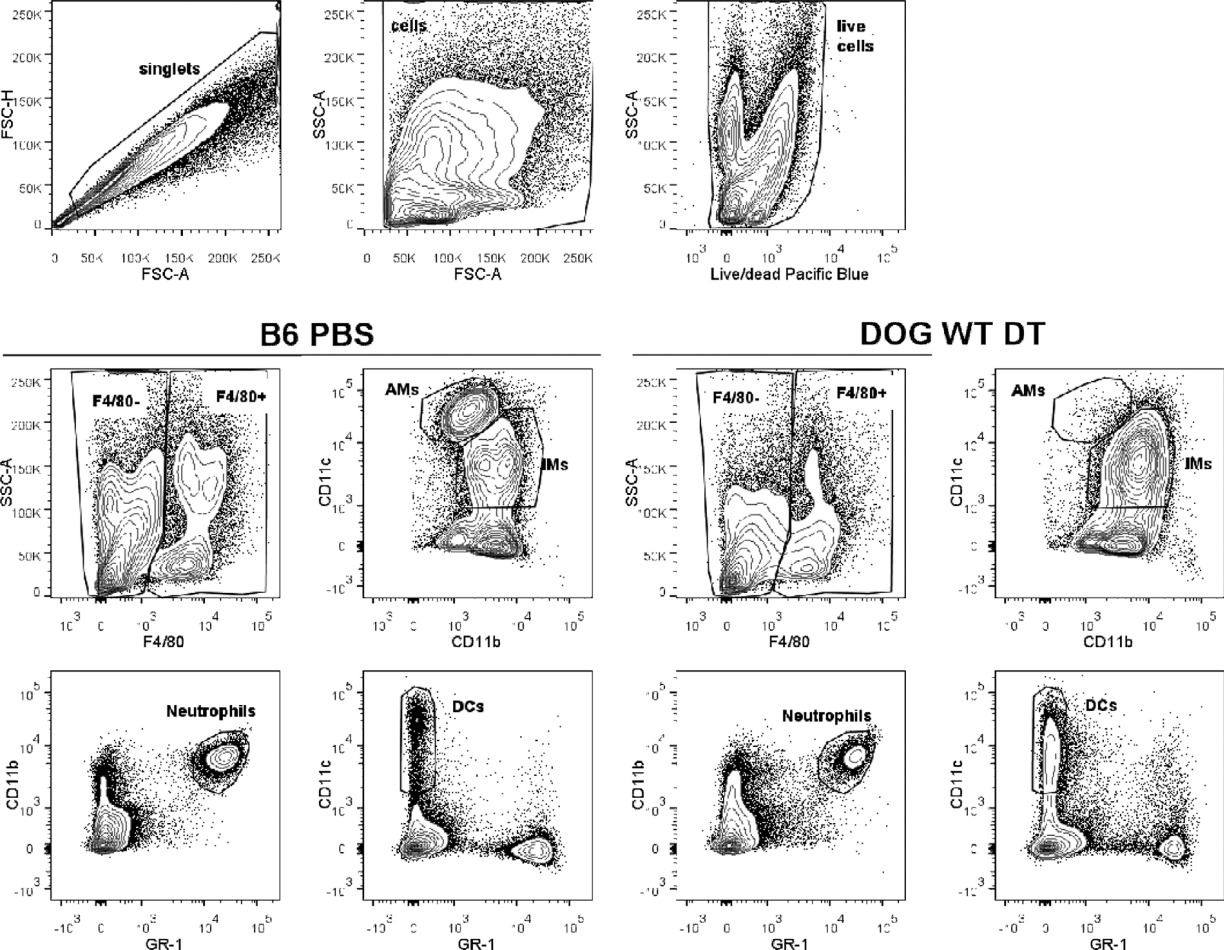
Flow cytometry gating scheme. Singlet cells were gated along the diagonal when forward scatter height (FSC-H) versus forward scatter area (FSC-A) was plotted. Cells were gated when side scatter area (SSC-A) versus FSC-A was plotted. Live cells were live/dead Pacific Blue negative when SSC-A was plotted versus live/dead Pacific Blue. F4/80^-^ and F4/80^+^ cells were discriminated by plotting SSC-A versus F4/80. From the F4/80^+^ gate, alveolar macrophages (AMs) were discriminated from interstitial macrophages (IMs) by plotting CD11c versus CD11b. From the F4/80- gate, neutrophils were identified by plotting CD11b versus GR-1 and dendritic cells (DCs) were identified by plotting CD11c versus GR-1. AMs were F4/80^+^, CD11c^+^, CD11b^mid^; IMs were F4/80^+^, CD11c^var^, and CD11b^+^; DCs were F4/80^-^, CD11c^+^, CD11b^-^; and neutrophils were F4/80^-^, CD11c^-^, CD11b^+^, and GR-1^+^. Contour plots are shown for both PBS treated B6 mice and WT DT treated DOG mice.

Cells were stained with 0.1 μg/mL Pacific Blue succimidyl ester (Life Technologies) to discriminate live and dead cells. Cells in a single-cell suspension had Fc receptors blocked with anti-CD16/32 (2.4G2) to prevent non-specific staining. Cells were then stained with CD3 AF488 (Clone 145-2C11, eBioscience), CD11b Pacific Blue (Clone M1/70, Biolegend), CD11c APC (Clone N418, eBioscience), CD19 PerCP-Cy5.5 (Clone 6D5, Biolegend), F4/80 PE (Clone BM8, eBioscience), and GR-1 AF700 (RB6-8C5, Biolegend). All antibodies were titrated on naïve B6 splenocytes and all experiments included fluorescence minus one controls. Samples were run on an LSRII (BDIS) and FlowJo10.06 (Treestar) was used for data analysis. Lung samples were gated as shown in Figure 6; a similar gating scheme was used for the spleen. In the lung, alveolar macrophages (AMs) were F4/80^+^, CD11c^+^, CD11b^mid^; interstitial macrophages (IMs) were F4/80^+^, CD11c^var^, and CD11b^+^; dendritic cells (DCs) were F4/80^−^, CD11c^+^, CD11b^−^; and neutrophils were F4/80^−^, CD11c^−^, CD11b^+^, and GR-1^+^. In the spleen, DCs were gated as F4/80^−^, CD11c^+^, and CD11b^−^ and neutrophils were gated as F4/80^−^, CD11c^−^, CD11b^+^, and GR-1^+^.

### Determination of Cytokine and Chemokine Concentrations

CD11c.DOG mice were intranasally inoculated with DT and B6 mice were intranasally inoculated with PBS. Five days later, mice were intranasally inoculated with PBS (mock) or 10,000 CFU *Francisella tularensis* LVS resuspended in 50 μL of PBS after being anesthetized with 575 mg/kg tribromomethanol (Avertin) (Sigma) administered intraperitoneally. Four hours later, mice were euthanized and lungs were homogenized using a Biojector (Bioject) and then clarified by centrifugation. A multiplex bead assay was used to determine cytokine and chemokine concentrations in lung culture supernatant as previously described 33. MCP-1 concentrations were determined in the lung homogenate using ELISA (Biolegend).

### Statistical Analysis

A Mann–Whitney was used to determine statistical differences in flow cytometry data and ELISA data when two groups were compared. An ANOVA with Tukey's post-test was used to determine whether B6 PBS, B6 WT DT, and B6 Mut DT were significantly different. GraphPad Prism (v5.04) was used for analysis. Error bars show standard error of the mean. Significance levels are indicated as follows: *P < 0.05; **P < 0.01, ***P < 0.001, ****P < 0.0001.
